# A Mass-Producible Washable Smart Garment with Embedded Textile EMG Electrodes for Control of Myoelectric Prostheses: A Pilot Study

**DOI:** 10.3390/s22020666

**Published:** 2022-01-15

**Authors:** Milad Alizadeh-Meghrazi, Gurjant Sidhu, Saransh Jain, Michael Stone, Ladan Eskandarian, Amirali Toossi, Milos R. Popovic

**Affiliations:** 1 The Institute for Biomedical Engineering, University of Toronto, Toronto, ON M5S 3G9, Canada; Milos.Popovic@uhn.ca; 2 KITE Research Institute, Toronto Rehabilitation Institute, University Health Network (UHN), Toronto, ON M5G 2A2, Canada; 3 Myant Inc., Toronto, ON M9W 1B6, Canada; gurjant.sidhu@myant.ca (G.S.); saransh.jain@myant.ca (S.J.); michael.stone1231@gmail.com (M.S.); ladan.eskandarian@mail.utoronto.ca (L.E.); amirali.toossi@myant.ca (A.T.); 4 Department of Mechanical and Mechatronics Engineering, University of Waterloo, Waterloo, ON N2L 3G1, Canada; 5 Department of Materials Science and Engineering, University of Toronto, Toronto, ON M5S 3E4, Canada

**Keywords:** electromyography, smart textiles, dry-contact electrodes, e-textile, conductive elastomeric yarn, knitted sensor, fabric sensor, ANN, prosthetic control

## Abstract

Electromyography (EMG) is the resulting electrical signal from muscle activity, commonly used as a proxy for users’ intent in voluntary control of prosthetic devices. EMG signals are recorded with gold standard Ag/AgCl gel electrodes, though there are limitations in continuous use applications, with potential skin irritations and discomfort. Alternative dry solid metallic electrodes also face long-term usability and comfort challenges due to their inflexible and non-breathable structures. This is critical when the anatomy of the targeted body region is variable (e.g., residual limbs of individuals with amputation), and conformal contact is essential. In this study, textile electrodes were developed, and their performance in recording EMG signals was compared to gel electrodes. Additionally, to assess the reusability and robustness of the textile electrodes, the effect of 30 consumer washes was investigated. Comparisons were made between the signal-to-noise ratio (SNR), with no statistically significant difference, and with the power spectral density (PSD), showing a high correlation. Subsequently, a fully textile sleeve was fabricated covering the forearm, with 14 textile electrodes. For three individuals, an artificial neural network model was trained, capturing the EMG of 7 distinct finger movements. The personalized models were then used to successfully control a myoelectric prosthetic hand.

## 1. Introduction

The overall goal of this work was to develop a scalable smart garment system capable of capturing muscles’ activity, controlling myoelectric prostheses or exoskeletons, and ultimately improve accessibility and functionality of assistive devices for individuals with amputation and mobility deficits. Mobility deficits are common debilitating outcomes of neurological injuries such as stroke and spinal cord injury, as well as limb amputation procedures. In 2013, the prevalence of stroke survivors worldwide was estimated to be 25,000,000 people [1]. In the United States alone, approximately 2 million people live with limb amputation [2]. Myoelectric prostheses and exoskeletons (further referred to as active prostheses) are wearable robotic prostheses that enable and assist users in performing motor functions they have lost due to amputation or neuromuscular deficits. The complexity of these prostheses vary depending on the user’s needs, the location and the level of amputation, or deficit. In this work, we aim to address some of the most common barriers to the adoption and frequent use of active prostheses in daily living conditions, by developing a smart garment with embedded textile electrodes.

Individuals with limb amputation report functionality limitations, discomfort, difficulty of usage, prosthetics’ heavyweight, and lack of breathability as top reasons for abandoning the usage of prostheses [3]. A common method for controlling myoelectric prostheses is to utilize electromyography (EMG) signals from users’ residual or intact limbs. Recent advances in machine learning pattern recognition algorithms have been improving the EMG-based control and functionality of myoelectric prostheses [4,5]. However, the limitations of neuromuscular interfaces for the comfortable and reliable acquisition of EMG signals in this context remain. Existing commercial systems currently use metallic electrodes or electrodes made of polymeric films to interface with the neuromuscular system which are not flexible or breathable [6,7], and as a result, are not comfortable for continuous use [3], and may contribute to skin complications [8].

Alternatively, in research settings, textile interfaces have also been explored for surface EMG recordings. The signal-to-noise ratio (SNR) of textile electrodes has been shown to be comparable to the gold standard EMG adhesive electrodes with gel [9,10]. Additionally, knitted textile electrodes are breathable and more comfortable for prolonged use, compared to existing metallic or gel adhesive neural interfaces, and have the potential to be incorporated in day-to-day clothing. Some systems also use textile contactless electrodes but have their own limitations such as low signal fidelity and are prone to higher noise [11,12,13]. Usability studies for smart textile systems for monitoring patients show a strong preference for comfort, unobtrusive, and light form factors that do not affect normal activities in daily life [14,15,16]. Bergmann et al. [15] mention that preferences clinicians have which aligned more with collecting and accessing data, limited storage for long-term recording, and the medical industry infrastructure [15]. Previous studies utilizing textile electrodes have also been successful in implementing algorithms including, linear discriminant pattern recognition algorithm and supervised pattern recognition algorithm, to differentiate between specific gross motor movements of the upper limbs [9,10,17]. However, textile EMG interfaces implemented so far are limited in their reusability and scalability (i.e., mass producibility) as they have utilized silver material yarns in their electrodes [18], which are not wash and dry resistant and are prone to sulfidation over time [17]. Among these studies, Farina et al. [19], Brown et al. [9], and Sumner et al. [20] had moistened the electrodes to achieve high fidelity recordings, which limits their application to only performing short-term recordings. The addition of moisture to the skin interface can also increase the chance of skin irritation and infection during prolonged use [8]. Lorussi et al. [21], developed a similar smart garment but used large electrodes with a comparably large inter-electrode spacing which reduces what muscles can be measured and the number of channels in an area. The works mentioned previously show the promising nature of e-textiles but neglect to cover one of the big barriers to commercialization which is washability and scalability [22], and to demonstrate functionality in real-world scenarios. The smart garments (i.e., sleeves) presented in this work build on the concepts of advanced additive manufacturing and 3D printing technologies by utilizing CAD-based automated knitting machines. Success in this development presents an important opportunity for the fabrication of highly customizable (CAD-based) EMG smart garments with significant mass-producibility potential. Similar to the familiar 3D printer example, mass production and transferability are realized by using automated knitting machines anywhere in the world along with the developed knitting programs. This is in contrast to previously published work on EMG-based smart garments, which are primarily proof of concepts, employing techniques with limited reproducibility in scale [22]. Seamlessly embedding electrodes with conductive textile traces for signal transmission is also important in an EMG smart garment to create a sleek and lightweight garment for patient usability. In the context of prosthetic limbs, a bulky system with many indwelling wires would not be practical. Previous works have not demonstrated seamless implementation of an EMG smart garment and have faced challenges such as electrical isolation in knitted fabrics [20,23]. The objective of this work was to address the outstanding technology gaps towards the needs of individuals with amputation and create an industry-scalable smart garment solution for EMG-based control of real-world myoelectric prostheses applications. Specifically, we developed a novel, dry textile electrodes and smart garments that are realizable using CAD-based knitting machines, provide excellent EMG signal fidelity, do not require indwelling wires for connecting to textile electrodes, are consumer wash cycle resistant (30×), and as such reusable, and finally can functionally be used for control of myoelectric prostheses in real-world applications with comfort. Additionally, to assess the limitations of the general use of the developed system and improve it in the next steps, testing was carried out with multiple volunteers.

## 2. Materials and Methods

A textile forearm sleeve was fabricated with an embedded array of textile electrodes. The textile electrodes were used for continuous recording of EMG signals from the forearm’s flexor and pronator muscles. An artificial neural network model was trained to distinguish various finger movements, and the resulting algorithm was embedded onto a microcontroller module to perform online recording, classification, and control of a myoelectric prosthetic hand.

**Figure 1:**
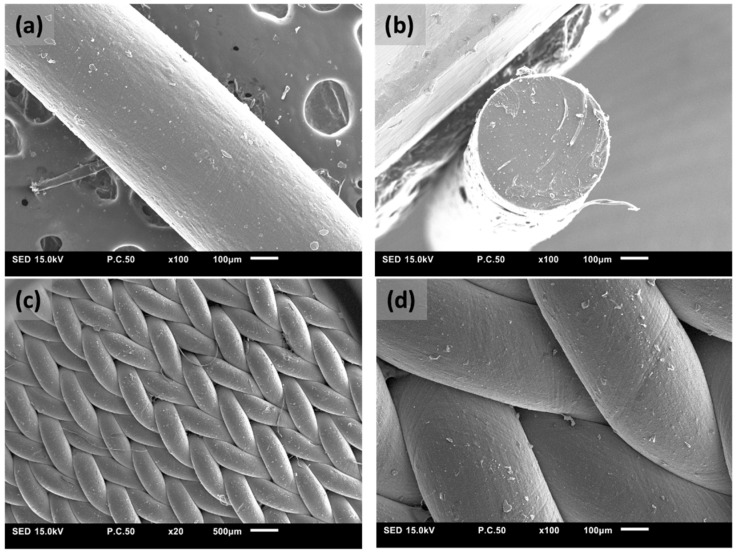
(**a**) Surface morphology of a conductive silicone rubber (CSR) yarn characterized by SEM; (**b**) Cross-section morphology of a CSR yarn; (**c**) Morphology of a dry textile electrode made of CSR yarn at 20× magnification; (**d**) Morphology of a dry textile electrode made of CSR yarn at 100× magnification.

Textile electrodes: Dry textile electrodes were knitted using conductive filament yarns made of carbon-contained silicone rubber (Myant Inc. in collaboration with University of Toronto, Canada). Silicone-based conductive yarns were chosen due to their mechanical robustness and flexibility for creating a conformal interface with the skin. Figure 1a,b show the surface and cross-section morphology of a conductive silicone rubber (CSR) filament yarn. Conductive yarns were then knitted into 3D structure textile electrodes using a flatbed knitting machine to provide and maintain consistent conformal contact with the skin (Figure 1c,d). JSM1000 SEM (JEOL, Akishima, Japan) was used to evaluate the morphological characteristics of CSR yarns and knitted electrodes.

Skin-Electrode Impedance Measurements: Skin-electrode impedance measurements were carried out on 3 study participants using an Ivium Vertex One potentiostat (Ivium Technologies, Eindhoven, Netherlands) in the galvanostatic mode in the frequency range of 1–100 kHz (5 freq/dec). Measurement protocols were according to those described by Spach et al. [24]. Textile and gel adhesive electrode samples used for these measurements were 2 cm × 2 cm (rectangular) and 2 cm in diameter (circular), respectively.

**Figure 2:**
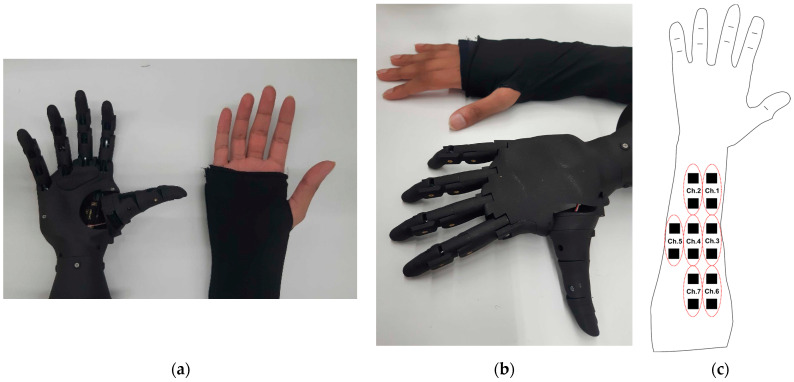
The myoelectric prosthetic hand and the EMG sleeve used in this study: (**a**) Top-view; (**b**) Side-view; and (**c**) location and arrangement of the textile electrode array with respect to users’ right forearm.

**Figure 3:**
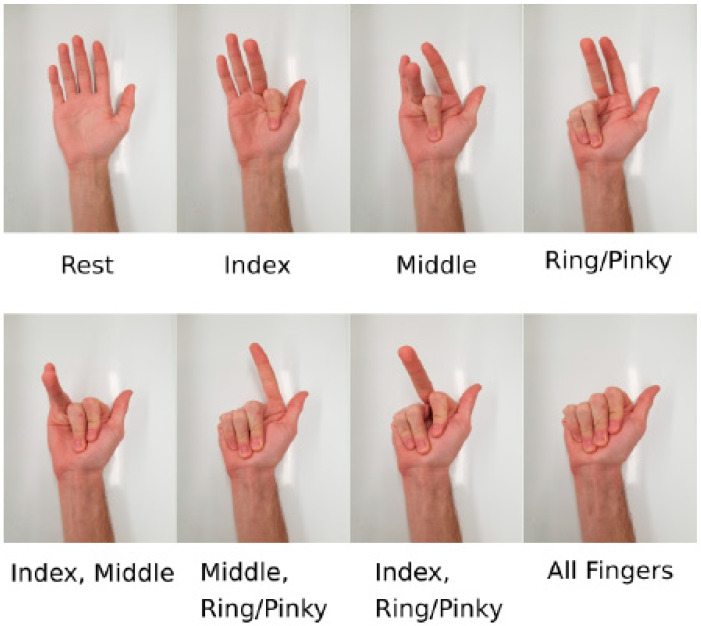
Hand gestures are used for the training of an artificial neural network (ANN) model.

Measurements for EMG Signal to Noise Ratio and Power Spectral Density Calculations: To calculate EMG signal to noise ratio (SNR) and power spectral density (PSD) for textile and gel electrodes, electrodes were positioned over the anterior surface of the forearm (position marked as channel 2 in Figure 2c). EMG signals were then recorded capturing 10 contractions (all finger flexion movement shown in Figure 3). The active state of an EMG was detected when the root mean square (RMS) amplitude of the EMG signal exceeded 6xSD of the baseline. SNR was calculated as the ratio of the RMS amplitude of the signal during active and inactive states. Power spectral density was calculated using Welch’s method.

**Figure 4:**
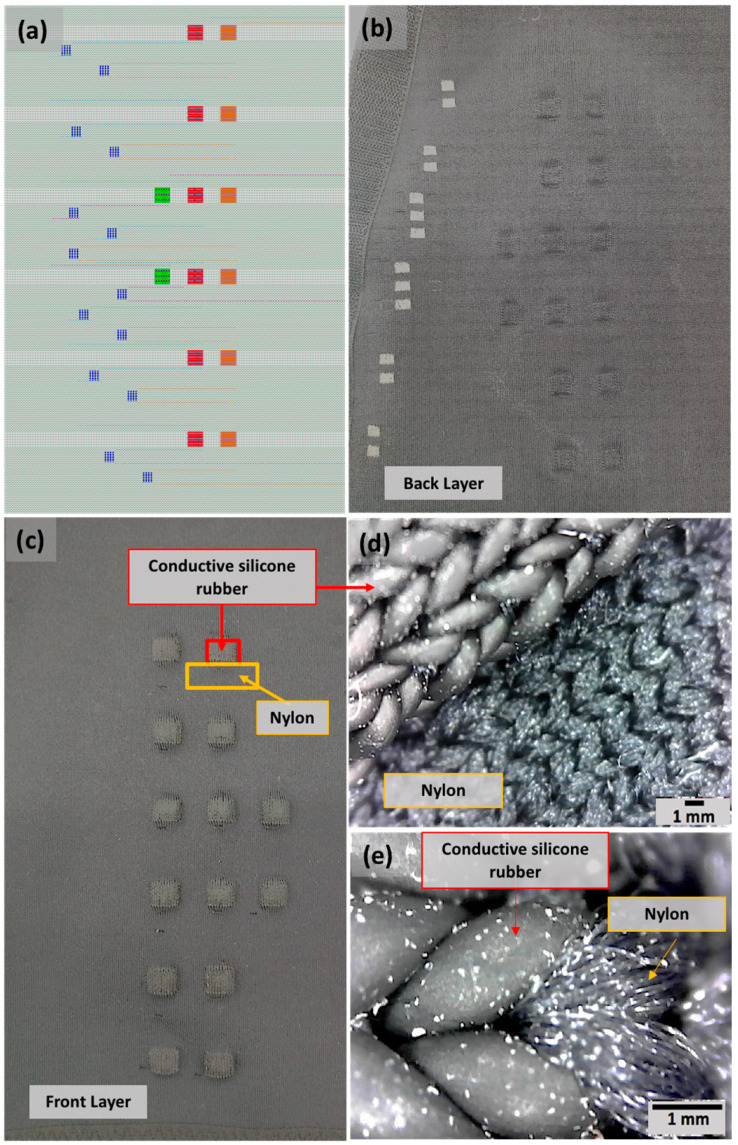
Textile sleeve with an embedded EMG electrode array: (**a**) Textile sleeve simulated fabric view; (**b**) Textile EMG sleeve outer view; (**c**) Textile EMG sleeve inner view; Optical microscope images showing seamless embedding of the electrode structure within a passive sleeve garment. During knitting, the textile electrode made of CSR yarns is directly connected to the electrically passive surrounding fabric made of nylon yarn (**d**) 40× magnification, and (**e**) 250× magnification.

EMG Sleeve (Multielectrode array): Fully integrated textile sleeves with 14 3D-structure textile electrodes were knitted using 18-gauge industrial flatbed knitting machines (Stoll, Reutlingen, Germany) (Figure 4b–e). 3D Knitted electrodes were positioned over the anterior forearm, extending over the pronator teres, flexor carpi radialis, palmaris longus, flexor carpi ulnaris, and flexor digitorum superficialis muscles which are responsible for wrist and finger flexion and pronation movements. A reference electrode was also positioned at the dorsal surface of the elbow joint. The surrounding fabric of the electrode had a double jersey structure made of Nylon (210 denier*) and Lycra (70 denier) yarns (Figure 4c–e). Silver-plated nylon yarn (100 denier) was used as conductive traces to connect the electrodes to the electronics, this yarn was hidden between the front and back layers to avoid being in direct contact with the body. The electrode sleeve (length: 35 cm, wrist circumference: 18–21 cm, elbow circumference: 28–31 cm) had velcro on the edges such that it could be securely fitted on the forearm of each test subject with a thumbhole to guide the placement and prevent shifting. Interelectrode spacing is defined as the center to center distance between the conductive area of 2 textile electrodes. The interelectrode spacing was 2 cm and 3 cm in mediolateral and longitudinal directions, respectively. The size of each 3D textile electrode was 1 cm × 1 cm. The spacing and size of electrodes were used to increase the density of recording sites on the forearm. Each pair of electrodes were assigned channels 1–7 (Figure 2c). This design was implemented to capture and distinguish seven finger flexion movements (Figure 4 shows the labeled hand gestures) using EMG classification, as a proof of concept.

EMG data acquisition and microcontroller unit: EMG signals were captured using a custom-built 8-channel data acquisition system (Myant Inc., Toronto, ON, Canada) consisting of a preamplifier stage and an analog input stage. Collectively the utilized EMG data acquisition system acquired EMG from 7 differential inputs with a sampling rate of 1 kHz and applied hardware filters to exclude frequency components outside of the 19–500 Hz range [25,26,27]. High-pass filter parameters were chosen based on previous literature to eliminate motion artifact components of the signal, inherent to EMG recordings [25,26,28,29]. The low-pass cutoff frequency was chosen since surface EMG signals are composed of 500 Hz signals and lower, so a cutoff of 500 Hz helps isolate EMG from high-frequency noise [27].

High-pass filtering at 19 Hz is achieved using a second-order Butterworth filter and low-pass filtering at 500 Hz was achieved using a third-order Bessel filter. Hardware filter implementations used in this study reduced the computation load of the microcontroller for feature calculation and control of the myoelectric prosthesis. An STM32F103RBT6 (STMicroelectronics, Geneva, Switzerland) microcontroller was used to perform online feature calculations, classification, and ultimately control of the myoelectric prosthetic. The microcontroller received input from the DAQ module (8 channels of filtered EMG) and provided output to the stepper motors of the myoelectric prosthetic. The firmware for this microcontroller was designed to calculate feature vectors in 270 ms bins and generate classifications based on an embedded artificial neural network (ANN) model (details provided in the subsequent sections). The firmware had two runtime modes: (a) training and (b) testing. In the training mode, feature vectors were calculated and sent to the computer using a serial port interface. This mode was used to save data associated with training sessions. In the testing mode, feature vectors were similarly calculated, followed by classification using the embedded ANN model, and followed by generating the relevant control signal outputs (pulse width modulated (PWM) signals) to the servo motors in the myoelectric prosthetic.

Myoelectric prosthesis: A 3D printed multi-articulating myoelectric hand was built. Specifically, we fabricated the Handi Hand developed by the Blinc Laboratory (University of Alberta, Edmonton, AB, Canada) [30]. The Handi Hand is an anthropomorphic prosthetic hand capable of all-natural degrees of freedom of a human hand except for lateral finger movements. One servo motor on each of the five fingers controls the flexion-extension of the fingers with an additional servo motor controlling the abduction/adduction of the thumb for a total of six servos [31]. The ANN model outputs a 3-bit output state vector which maps to particular hand gestures that servo motors move to replicate (Table 1).

ANN Model Training: ANN models have traditionally been used for real-time gesture classification of surface EMG signals [32,33] and were also utilized in this study. To train an artificial neural network, three EMG signal time-domain features were chosen [34,35]: (i) Willison amplitude (WAMP) [36], (ii) Wavelength (WL) [35], and (iii) Root Mean Square (RMS).

The WAMP feature is related to the number of motor unit action potentials (MUAPs) which occurred during the effective window [17] and is defined as

(1) $$\mathrm{WAMP}=\overset{N}{\underset{i=2}{\sum }}\;f\left(\left|x_{i}-x_{i-1}\right|\right)$$

where:

$x_{i}$ is the value of the *i*-th sample of data within the effective window

N is the number of samples within the effective window

f(x) is a function equal to 1 if x>ε and equal to 0 otherwise, and

ε is some arbitrary threshold value

The WL is a measure of the complexity of the signal [37] and increases with the strength of an EMG response. WL is defined as

(2) $$\mathrm{WL}=\overset{N}{\underset{i=2}{\sum }}\;\left|x_{i}-x_{i-1}\right|$$

where:

$x_{i}$ is the value of the *i*-th sample of data within the effective window

N is the number of samples within the effective window

RMS is a popular feature for EMG analysis and has been shown to be correlated with muscle contraction force [38]. RMS is defined as

(3) $$\mathrm{RMS}=\sqrt{\frac{1}{N}\overset{N}{\underset{i=1}{\sum }}\;x_{i}^{2}\;}$$

where:

$x_{i}$ is the value of the *i*-th sample of data within the effective window

N is the number of samples within the effective window

**Figure 5:**
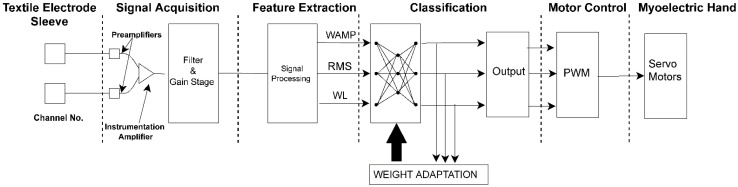
Flowchart of the system used for the EMG-based control of the myoelectric prosthetic hand shown for one of the seven channels.

For each study participant, a multilayer feedforward artificial neural network (ANN) model with back-propagation and mini-batch gradient descent was trained to classify seven finger flexion movements (Figure 3). EMG recordings were done using seven channels (Figure 2c). The ANN models were trained with one hidden layer, three hidden neurons, a learning rate of 0.0001, and a batch size of five to train 6000 epochs. The training was done using a custom-written MATLAB program (Ver. 9.10.0.1602886 (R2021a), MathWorks, Natick, MA, USA). A total of 5 datasets were used to model training and another 5 datasets were used to evaluate the classification accuracy of the model. Each participant had a personalized ANN model trained using only their data. The resulting trained model was then embedded onto the microcontroller to perform live classification and control the myoelectric prosthesis. The full system block diagram which shows how each part is integrated can be seen in Figure 5.

Personalized ANN Model Developed: Personalized models are versatile solutions that account for natural variabilities in individuals’ anatomy. This is in contrast with the development of a single universal model that can be used on a large population of users. This is especially the case in those with amputated limbs where the morphology of the residual limb is variable [33,34]. The necessity of a personalized solution for individuals with amputation is well known and commonly implemented in areas such as a prosthetic socket design and fabrication and motor control strategies. Using this concept, every user trains with their smart garment only the first time. The concept of developing personalized models and calibration stages has also been demonstrated in various forms in existing commercial systems such as the Coapt engineering system [6].

Experimental Protocol: All procedures and protocols were approved by the research ethics board committee of the University of Toronto. Three volunteers (age: 26–28, male, right-handed, Table 2) participated in this study. All participants took part in one training and one test session. During the training sessions, participants donned the multi-electrode EMG sleeve on their right forearm (Figure 2c) or received an array of gel electrodes in the same locations, and were asked to perform all finger movement classes (Figure 3) in sequence multiple times. Specifically, the sequence starts with ten seconds of rest while data is collected for calibration purposes, followed by the participant holding each hand pose for 5 s, followed by a 10 s rest period in between. Each complete cycle of all hand poses formed one dataset. A total of 10 datasets were collected from each participant (5 for training and 5 for testing). At the end of each training session, the quality of the collected data was assessed to ensure that the test subject held each hand gesture for the correct time interval and each state could clearly be labeled for the training data.

In testing sessions, the trained ANN models, personalized to each test subject, were embedded in the microcontroller unit of the system, to perform online EMG-based classification of the finger movements. In these sessions, participants donned the EMG sleeves and using their embedded trained model attempted to control the myoelectric prosthesis to replicate the movements of their hands. In each session, participants were asked to perform each hand pose at least 10 times. The success rate of the system in controlling the myoelectric hand with specific finger movements was quantified and assessed on video.

Washing Cycle: Given the objective of this work in presenting a scalable and reusable solution for EMG-based control of myoelectric prosthesis, the effect of consumer garment wash cycles was also investigated for 30× washes which is a common industry target for the robustness of the quality of clothing [17]. Textile electrode swatches (n = 3) were washed 30 times according to the American Association of Textile Chemists and Colourists (AATCC) home laundry washing test method using a commercial washing machine (Whirlpool WED5600X) under a normal laundry cycle for a small load with cold water using AATCC Standard Reference Detergent Without Optical Brightener (SDL Atlas, Rock Hill, SC, USA). Electrode swatches and EMG sleeve samples were placed in a mesh laundry bag during laundering. After each laundering cycle, samples were laid flat and left to dry at room temperature prior to the next wash cycle. Washed electrode swatches were then compared with unwashed swatches and EMG sleeves in terms of their EMG signal-to-noise ratio and classification accuracy [17].

Data Analysis: After each training session, the collected EMG data were labeled and an ANN model was trained, and a confusion matrix was generated using a custom-written MATLAB program. The confusion matrix was calculated by comparing the feature vectors of the test data into the ANN model with expected outputs. Testing sessions’ data was acquired by quantifying the participants’ hand poses and the resulting prosthetic hand poses on video. This data was then used to generate confusion matrices using a custom-written MATLAB program. Signal-to-noise ratio (SNR) calculations of the EMG signal were defined as the ratio of the RMS of the signal during a bout of EMG activity (during muscle contraction) divided by the RMS of the background activity (no muscle contraction). All SNR calculations were also done using a custom-written MATLAB program. All statistical analyses were done using GraphPad Prism 8 software (GraphPad Software, San Diego, CA, USA). Comparisons between the SNRs of textile and gel electrodes, and those of unwashed vs. 30× washed textile electrodes, were carried out using unpaired t-tests. Normality was tested using the Shapiro–Wilk tests.

## 3. Results

Electrode Design Evaluation: To create 3D structure textile electrodes using CSR yarns, three layers were knitted and seamlessly integrated: (1) the surface of the electrode was made of CSR yarn, (2) nylon yarn was used as a spacer layer which was knitted under the surface layer acting as a filler to create a 3D raised structure, and (3) nylon yarn was also used to knit the back layer to provide support to the entire structure (Figure 4d). This 3D knit structure of the electrode allowed for more intimate contact between the electrode and the user’s body during various activities in the limbs that create dynamic movements in the muscles, and a change in the skin-electrode contact. This issue is not noticeable with gel electrodes, due to the adhesive layer that ensures intimate contact with the skin and traditional flat textile electrodes do not have this feature. In addition, the automated knitting process was used to create a highly dense and homogenous conductive surface (Figure 4c–e). The integration of the textile electrodes with the rest of the garment was done seamlessly (Figure 4e) with the passive garment. During knitting, the textile electrode made of conductive silicone rubber was directly connected to the electrically passive surrounding fabric made of nylon yarn. Additionally, using the electrically passive fabric properties and the feasible distance between two yarn feeders the minimum distance between the two conductive traces was optimized to achieve electrical isolation. Finally, participants were interviewed after wearing the sleeves with embedded electrodes for this study and found it to be comfortable on the skin when worn and the entire design to be lightweight and practical.

**Figure 6:**
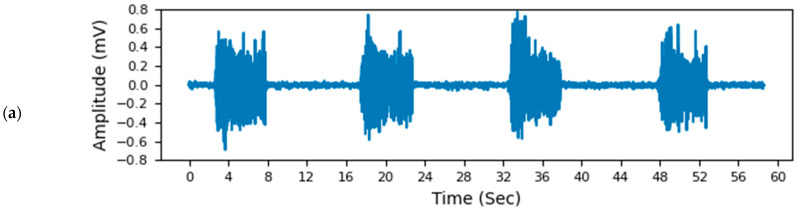

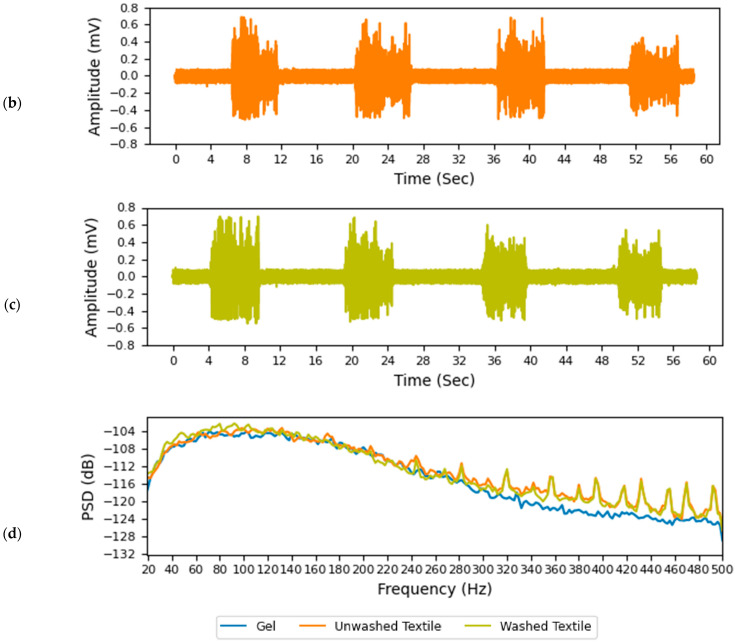
EMG signals collected on channel 2 (Figure 3c) of EMG Textile Sleeve during all finger flexion hand pose using. (**a**) Gel electrodes (2 cm diameter) and (**b**) Unwashed Textile Electrode to compare textile electrodes SNR against the gold standard. (**b**,**c**) demonstrate the EMG signals collected using the same methods on unwashed (**b**) and 30-times washed (**c**) textile electrodes to assess the possible effects of washing cycles on SNR. (**d**) shows the PSD plots (**a**–**c**) of gel electrodes, unwashed textile electrodes, and washed textile electrodes. The *x*-axis range of the PSD plots represents the frequency range of the filtered signal (19–500 Hz).

**Figure 7:**
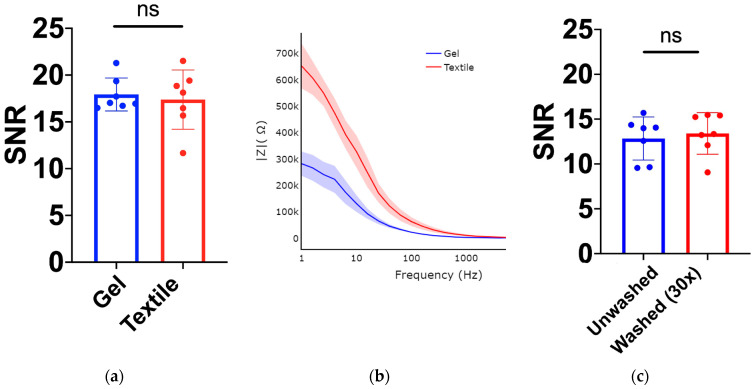
SNR calculations on EMG signal collected on Ch. 2 of EMG textile sleeve (Figure 2c channel locations) during all finger flexion hand pose. ‘ns’ represents ‘not a statistically significant difference’ between the groups. Bar graphs represent the standard deviation of the mean. (**a**) Gel Electrode (2 cm diameter) vs. textile electrode swatch (2 cm × 2 cm), (**b**) Skin-electrode Impedance plot for unwashed textile electrode (2 cm × 2 cm) and gel electrode (2 cm diameter), (**c**) Unwashed textile electrode swatch (2 cm × 2 cm) vs. Washed textile electrode swatch (2 cm × 2 cm).

**Figure 8:**
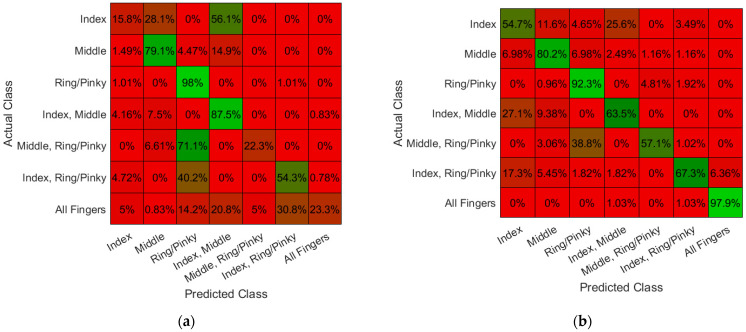
Offline classification performance and the associated confusion matrices of the trained models for finger gesture detection: (**a**) Offline classification with Gel Electrodes; (**b**) Offline classification with Unwashed EMG Textile Sleeve.

Performance of Textile Electrode Against Gold Standard: The machine-knitted electrodes with an array of embedded dry textile electrodes were successful in acquiring high fidelity EMG signals (Figure 6). The signal fidelity of the textile electrodes was evaluated by comparing the EMG signal of 2 cm × 2 cm textile electrode swatches with similar size gel electrodes (2 cm diameter). SNR of the EMG signals acquired from textile electrodes swatches (17.4 ± 3.2, mean ± standard deviation) was found to be comparable (Figure 7a, *p* = 0.69) to those acquired using gel adhesive electrodes (17.9 ± 1.8). The power spectral densities (PSDs) of the EMG signals were also found to be highly correlated between the gel electrodes and textile electrode swatches (correlation coefficient of 0.97 (Figure 6d). Dry textile electrodes were found to have a larger impedance than that of the gel adhesive electrodes (Figure 7b). At 250 Hz impedances were 10.8 ± 0.9 kΩ for gel, and 32.2 ± 9.6 kΩ (*p* = 0.198). To further compare the performance of textile electrodes with that of the gel electrode, ANN models were trained using a gel electrode array as well as a textile EMG sleeve. The EMG sleeve electrode size was reduced by 1 cm × 1 cm compared to the textile electrode swatches of 2 cm × 2 cm in size to increase the spatial density of textile electrodes on the forearm. Confusion matrices of the developed models are shown in Figure 8. The model generated for the gel electrode array was on average 54% successful in classifying the gestures. In contrast, the textile sleeve was on average 73% successful in classifying the gestures. The superior performance of the textile electrodes distinguishing different classes may be due to their smaller relative dimensions (1 cm × 1 cm for EMG sleeve electrodes compared to 2 cm diameter for off-self gel electrodes) [39].

**Figure 9:**
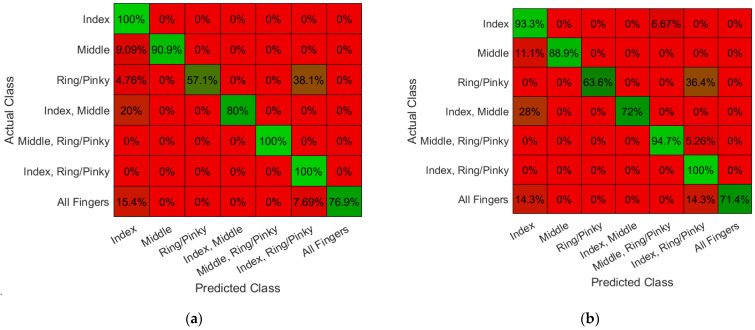
Online Classification performance and the associated confusion matrices of the trained models for finger gesture detection: (**a**) Online Classification with Unwashed EMG Textile Sleeve; (**b**) Online Classification with 30× Washed EMG Textile Sleeve.

Effect of Consumer Wash Cycle on the Performance of the Proposed Smart Textile: Textile electrodes were found to be resistant to the consumer wash cycle process (SNR unwashed 12.8 ± 0.9), as the EMG signal SNR did not deteriorate even after 30× wash cycles (SNR washed 13.4 ± 0.88) (Figure 7c, *p* = 0.66). The correlation coefficient of the PSD (Figure 6d) between unwashed textile electrode swatches and washed textile electrode swatches was 0.98. The correlation coefficient between washed textile electrode swatches and gel was 0.97. To further assess the potential impact of the wash cycles on the classification and prosthetic control performance of a textile electrode array, an ANN model was trained using a textile EMG sleeve. The results of the online classification tests using the sleeve are shown in Figure 9a. The average classification success rate was found to be 86%. The same model was then used to test the sleeve after 30 consumer wash cycles and the online classification testing results are shown in Figure 9b. The average classification success rate post-wash was found to be 83%. These results also confirm that the proposed novel textile electrode arrays are not degraded by the wash cycle and the proposed smart textile can be reused by the user to achieve similar outcomes in real-time control of myoelectric prosthetics.

**Figure 10:**
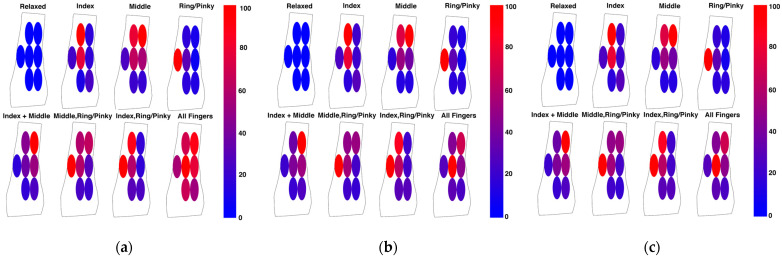
Normalized distribution of feature vectors for different channels during specific hand gestures: (**a**) Willison Amplitude (WAMP); (**b**) Root mean square (RMS), and (**c**) Wavelength (WL).

**Figure 11:**
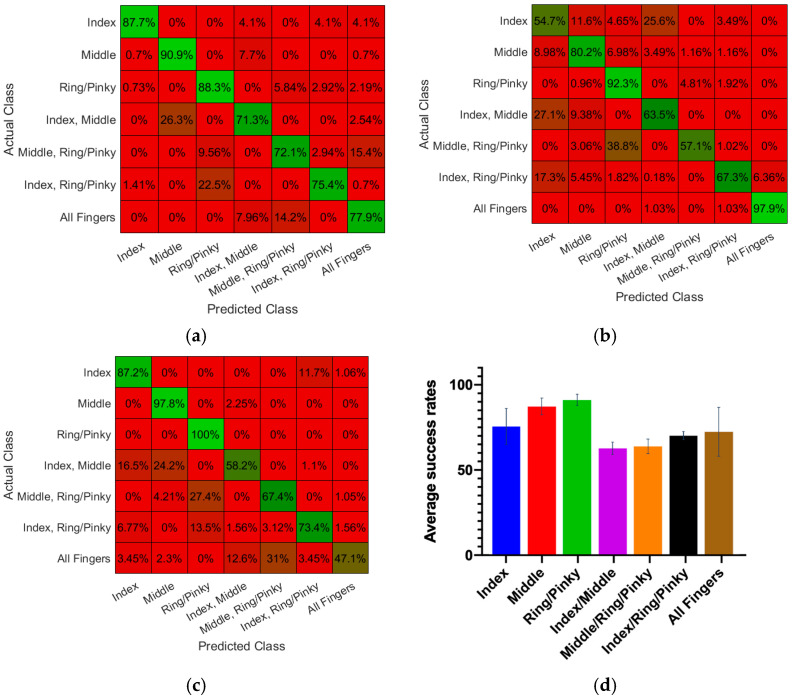
Offline classification performance and the associated confusion matrices of the trained models for finger gesture detection: (**a**) Test Subject 1; (**b**) Test Subject 2; (**c**) Test Subject 3, and (**d**) Average success rates of the trained models in gesture classification across all test subjects.

Finger Gesture Classification and Real-time Control of a Myoelectric Prosthetic using the Proposed Smart Textile: The chosen feature vectors for training an ANN model using the textile EMG sleeve produced distinguishable activity patterns for the targeted finger poses. Figure 10a–c, shows normalized distributions of the feature vectors. Trained ANN models had an average overall classification success rate of 81%, 73%, and 76% for three participants of the study. The resulting training confusion matrices are shown in Figure 11. The specific average classification success rates for *Index*, *Middle*, *Ring/Pinky*, and *all fingers flexion* movements, were 77%, 90%, 94%, and 74%, respectively. The average classification success rates for *Index + Middle*, *Middle + Ring/Pinky*, and *Index + Ring/Pinky* movements were 64%, 66%, and 72%, respectively.

**Figure 12:**
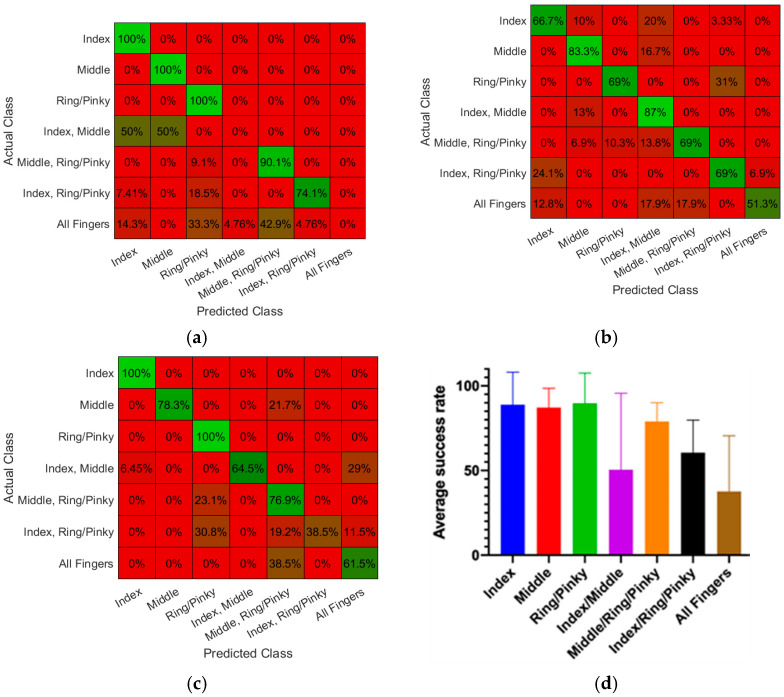
Results of testing the performance of the model implemented for online classification of the gestures: (**a**) Confusion matrix plots for tests with subject 1, (**b**) Confusion matrix plots for tests with subject 2, (**c**) Confusion matrix plots for tests with subject 3, and (**d**) Average success rate of the model in the online classification of the gestures across all test subjects.

The trained ANN model was then embedded onto the data acquisition and microcontroller module and tested for online classification and control of the myoelectric prosthetic hand. The experimental setup and testing process is shown in Supplementary Video S1. The firmware was C-compatible. uVision (Ver.5.34.0.0, Keil, Grasbrunn, Germany) IDE, which has embedded libraries for the STM microcontroller, was used to commit the ANN model to the board. The feature vectors are calculated in bins and sent to the microcontroller actuating the hand at a rate of 270 ms, which allows close to a real-time response. Having these bins reduces the computational load on the board, as only 270 samples of data are saved at a time. All three study participants were tested using their developed ANN models and the results are shown in Figure 12. The specific average testing success rates for *Index*, *Middle*, *Ring/Pinky*, and *all fingers flexion* movements, were 89%, 87%, 90%, and 38%, respectively. The average classification success rates for *Index + Middle*, *Middle + Ring/Pinky*, and *Middle + Ring/Pinky* movements were 50%, 79%, and 61%, respectively.

## 4. Discussion

Results obtained in this work demonstrate the feasibility of using a fully textile machine knitted interface for capturing EMG activity patterns and controlling myoelectric prostheses. Unlike previous works that utilized moist textile interfaces for high-fidelity EMG recordings [10,30], here we demonstrated that our proposed dry textile electrodes made of conductive silicone fibers provide recordings with SNRs comparable to that of the gold standard gel adhesive electrodes. Additionally, to our knowledge, this work presents the first evidence for EMG interfaces that are resistant to repeated standardized consumer wash cycles. Collectively, the high signal fidelity, breathability, dry interfacing, mechanical flexibility, as well as washability and reusability of the proposed textile EMG electrode arrays positions them as a promising solution to address the discussed limitations of the conventional neural interfaces used for continuous control of myoelectric prostheses. Additionally, the success of the machine knitted designs presented in this study, as opposed to hand-sewn designs and implementations, is suggestive of the industrial scalability and commercial potential of this solution. We also demonstrated a proof-of-concept ANN model for the online classification of finger movements based on forearm EMG activity. Comparing the success rates of the training phase and online classification testing suggests a similar trend among the three test subjects with single finger flexion movements having higher detection percentages than flexion synergies involving multiple fingers. Misclassification of synergistic movements that did occur were often detected as one of the movements making up the synergy. A possible reason for these misclassifications is due to the insufficient number and density of recording channels to distinguish synergistic movements from individual flexions. Future studies will build upon the findings presented in this work and increase the number, density, and coverage of the channels on both the anterior and posterior of the forearm. Additionally, this study used the same size sleeve with an adjustable enclosure mechanism to fit a wide range of forearm ranges (25–32 cm). While in the original design the interelectrode spacing was 1 cm in the mediolateral direction and 2 cm longitudinally, these values may have been affected (due to fabric stretch) when individuals with larger forearms used the sleeve). For this reason, test subjects with larger forearms (test subject 1) performed better than test subjects with smaller circumference forearms (test subject 2 and test subject 3). This limitation will be addressed by creating multiple sleeve sizes to homogenize the condition and relative density of the electrode across a large range of users

Other limitations that were identified in this work that will be tackled as next steps to the presented research: (1) Specific arrangement and position of the electrodes on individuals’ forearms is a critical consideration that was achieved by muscle palpitations and markings of anatomical landmarks in this work. To improve the general use and scalability of EMG sleeves, the implementation of garment alignment features will be pursued. Additionally, algorithms will be developed to select the best channels based on the calibration phase, gestures users would perform. (2) In this study, EMG sleeves were knit to size for the study participants, future work will also focus on developing standardized sizes based on anatomical landmark location estimates. This will also allow for a standardized electrode contact pressure. (3) This work presented a proof-of-concept ANN for the classification of finger movement. We implemented a personalized approach for the control of myoelectric prosthesis and developed ANN models for each study participant (user). Validation of this approach in this pilot study was limited to testing in only 3 participants and as such generalizability of this approach needs to be further investigated in future studies.

Finally, the application of wearable EMG interfaces such as the one presented here goes beyond prosthetic control and can be implemented for rehabilitation [40], accessible interfacing with computers and smartphones [41], as well as pain management such as mirror feedback therapy based treatments of phantom limb pain [42]. Future work in the field of smart textiles requires more comprehensive usability studies for a wider range of the population [43].

More importantly in the context of creating solutions for daily living conditions, proposed electrodes are resistant to repeated consumer wash cycles. To our knowledge, this is the first demonstration of a ‘consumer’ wash cycle safe (up to at least 30 times) smart garment for EMG-based myoelectric control and rehabilitation technologies, which is essential for long-term use in these applications.

## Figures and Tables

**Table 1:** Outputs state vector mapping of the classified gestures.

	Output State Vector		
Gesture	a	b	c
Rest	0	0	0
Index	0	0	1
Middle	0	1	0
Ring/Pinky	1	0	0
Index/Middle	0	1	1
Middle/Ring/Pinky	1	1	0
Index/Ring/Pinky	1	0	1
All Fingers	1	1	1

**Table 2:** Anthropometric Data.

Test Subject	Sex	Age	Forearm Circumference	Forearm Length	Dominant Hand	BMI	Height
1	Male	27	32 cm	27 cm	Right	27.6	6′2″
2	Male	27	25 cm	26 cm	Right	20.2	5′6″
3	Male	28	27.5 cm	27 cm	Right	22.8	5′8″

## Data Availability

Not applicable.

## Supplementary Materials

The following supporting information can be downloaded at: https://www.mdpi.com/article/10.3390/s22020666/s1, Supplementary Video S1: This video shows the proposed EMG recording and control system in action when the described textile sleeve was used to control a handihand myoelectric prosthetic.
